# Differential Methylation at Novel Putative Imprinting Control Regions on Chromosome 20 Associated with Childhood Obesity

**DOI:** 10.64898/2026.09.02.26362065

**Published:** 2026-09-07

**Authors:** Catherine F. Everly, Terrence K. Allen, David A. Skaar, Bruce A. Corliss, Dereje D. Jima, Rachel L. Maguire, Halah Jadallah, Randy L. Jirtle, Susan K. Murphy, Jung-Ying Tzeng, Cathrine Hoyo

## Abstract

DNA methylation may link early-life exposures to later obesity risk; however, most studies examine individual CpG sites using commercial arrays that cover <5% of the genome and often overlook biologically-relevant regulatory regions. Imprinting control regions (ICRs) regulate parent-of-origin gene expression and are enriched for growth and metabolic genes, representing strong candidates for developmental programming of obesity. In 586 participants of the Newborn Epigenetics STudy (NEST), we leveraged the novel Human Imprintome Array to measure methylation at >1,000 characterized and putative ICRs genome-wide in umbilical cord blood. We applied principal component and kernel machine regression to identify ICR methylation at birth associated with sustained childhood obesity, defined as BMI ≥95 ^th^ percentile during most of childhood. Sustained childhood obesity was associated with differential methylation at seven ICRs in cord blood, with 2-10% mean differences between obesity and normal weight groups, including novel regions localized to chromosome 20q11.1-20q11.21 (ICR_1182, ICR_1179, ICR_1181, ICR_1177, ICR_1180, and ICR_1165), mapping to or near *CDC27P4, LINC01597, DUX4L37,* and *DUX4L34,* and ICR_927 mapping to previously characterized *ZNF597/NAA60.* Most ICRs overlapped multiple transcriptional regulator binding sites. Associations persisted in peripheral blood collected in later childhood at ages 8-16 years. Among offspring of mothers with pre-pregnancy obesity, an additional 50 associations were identified. Sex-stratified analyses revealed no further insights. These findings identify novel ICRs—particularly on chromosome 20q—as potential early-life epigenetic biomarkers of childhood obesity. If replicated, these stable ICR methylation patterns may identify children at risk for obesity at birth, informing targeted prevention before obesity develops and guiding novel therapeutic strategies.

## Full Text Availability

The license terms selected by the author(s) for this preprint version do not permit archiving in PMC. The full text is available from the preprint server.

